# Metformin and vitamin D modulate adipose-derived stem cell differentiation towards the beige phenotype

**DOI:** 10.1080/21623945.2022.2085417

**Published:** 2022-06-23

**Authors:** Sara Cruciani, Giuseppe Garroni, Renzo Pala, Donatella Coradduzza, Maria Laura Cossu, Giorgio Carlo Ginesu, Giampiero Capobianco, Salvatore Dessole, Carlo Ventura, Margherita Maioli

**Affiliations:** a Department of Biomedical Sciences, University of Sassari, Sassari, Italy; b General Surgery Unit 2 “Clinica Chirurgica” Medical, Surgical and Experimental Sciences Department, University of Sassari, Sassari, Italy; c Department of Medical, Surgical and Experimental Sciences, Gynecologic and Obstetric Clinic, University of Sassari, Sassari, Italy; d Laboratory of Molecular Biology and Stem Cell Engineering, National Institute of Biostructures and Biosystems - Eldor Lab, Innovation Accelerator, Consiglio Nazionale delle Ricerche, Bologna, Italy; e Center for Developmental Biology and Reprogramming (CEDEBIOR), Department of Biomedical Sciences, University of Sassari, Sassari, Italy

**Keywords:** Adipose-derived stem cells, differentiation, cellular mechanisms, gene expression, adipogenesis, conditioned media

## Abstract

Adipose-derived stem cells (ADSCs) represent an ideal stem cell population for regenerative medicine. ADSC adipogenic differentiation is controlled by the activation of a specific transcriptional program, including epigenetic factors and key adipogenic genes. Under certain conditioned media, ADSCs can differentiate into several phenotypes. We previously demonstrated that bioactive molecules could counteract lipid accumulation and regulate adipogenesis, acting on inflammation and vitamin D metabolism. In the present paper, we aimed at evaluating the effect of metformin and vitamin D in targeting ADSC differentiation towards an intermediate phenotype, as beige adipocytes. We exposed ADSCs to different conditioned media and then we evaluated the levels of expression of main markers of adipogenesis, aP2, LPL and ACOT2. We also analysed the gene and protein expression of thermogenic UCP1 protein, and the expression of PARP1 and the beige specific marker TMEM26. Our results showed a novel effect of metformin and vitamin D not only in inhibiting adipogenesis, but also in inducing a specific ‘brown-like’ phenotype. These findings pave the way for their possible application in the control of *de novo* lipogenesis useful for the prevention of obesity and its related metabolic disorders.

## Introduction

Adipose-derived stem cells (ADSCs) represent a promising source of mesenchymal stem cells (MSCs) for tissue repair and regeneration **[**1**]**. ADSCs show high plasticity and immunomodulatory properties, being able to differentiate into several phenotypes, thus representing an excellent candidate in regenerative medicine approaches **[**2–4**]**. ADSC adipogenic differentiation is controlled by the activation of a specific transcriptional program, involving several transcription and epigenetic factors, including miRNAs **[**5**]**. There are several adipocyte-specific genes that are activated during adipogenesis **[**6**]**. Fatty acid binding protein (FABP) **[**4**]**, also known as aP2, is a lipid binding protein that acts as an adipokine in regulating systemic metabolism **[**7,8**]**. Lipoprotein lipase (LPL) is the primary enzyme involved in fatty acid uptake from lipoproteins and *de novo* lipogenesis **[**9**]**. In addition, LPL is closely related to cell growth and seems to be important for brown adipose tissue activation **[**10–12**]**. Also Acyl-CoA thioesterase 2 (ACOT2) plays a role as an auxiliary enzyme supporting efficient fat burning by a thermogenic mechanism **[**13–15**]**. Adipose tissue plays a key role in regulating metabolism and insulin sensitivity **[**16**]**. White adipose tissue (WAT) stores energy as fat depots and produces adipokines and prostaglandins **[**17,18**]**. Brown adipose tissue (BAT) dissipates heat as a result of fat metabolism and high mitochondrial activity **[**19**]**. Beige or brite adipose tissue shows strong mitochondrial activity, and derives from *de novo* adipocyte differentiation of stem and progenitor cells, or from a transdifferentiation of white adipocytes following a process called ‘browning’ **[**20,21**]**. Uncoupling protein 1 (UCP1) expressed by thermogenic adipocytes is critical for adaptive thermogenesis **[**22**]**. Elevated UCP1 levels contribute to the enhancement of basal glucose uptake in adipocytes **[**23**]**. Furthermore, manipulation of UCP1 expression has been shown to reduce obesity improving insulin sensitivity **[**24**]**. As well as brown adipocytes, beige cells respond to adrenergic stimuli through mitochondrial biogenesis, UCP1 expression, fatty acid degradation and heat generation **[**25**]**. In addition, beige adipocytes can be distinguished from other cell types thanks to specific markers, including PAT2 and TMEM26 **[**26,27**]**. By regulating adipocyte function and body energy balance, also Poly(ADP-Ribose)Polymerase-1 (PARP1) plays a role in obesity and obesity-related disorders **[**28**]**. PARP enzymes, upregulated during adipocyte development, are involved in the regulation of inflammation, increasing SIRT1 activity, and modulating PPARγ expression **[**29,30**]**. A surplus in energy storage, as in obese patients, leads to the release of inflammatory cytokines and adipokines, degenerating into a wide range of disorders, as cardiovascular diseases **[**31**]**. Several signalling pathways and epigenetic factors contribute to the regulation of MSC differentiation into specific phenotypes **[**32**]**. Moreover, it is largely demonstrated that many bioactive molecules regulate adipogenic differentiation and expression of key transcription factors **[**33**]**. Vitamin D is well-known for its ability to counteract adipogenesis while inducing osteogenesis **[**34,35**]**. Moreover, overweight and obesity are often associated with Vitamin D deficiency **[**36**]**. Within this context, we previously demonstrated that the combination of vitamin D and metformin is able to counteract ADSC adipogenic differentiation, modulating vitamin D metabolism and the expression of specific epigenetic factors **[**37,38**]**. In the present paper, we aimed at evaluating the capability of these two molecules in orchestrating stem cell differentiation towards the beige phenotype, with particular attention to the main adipogenic markers and UCP1 expression, as a potential therapeutic strategy to counteract obesity.

## Materials and methods

### Cell isolation and culturing

ADSCs were isolated from abdominal subcutaneous adipose tissue of men and women (n = 6, age = 45 ± 15 years, BMI: 22 ± 3 kg/m2) after acceptance and signing of informed consent. The study was approved by the Review Board of the Human Studies Ethics Committee of Sassari. Briefly, the tissue was washed in PBS (Euroclone, Milan, Italy; ref. ECB4004L) and digested by Collagenase type I solution (Gibco Life Technologies, Grand Island, NY, USA, ref. 17,010–029) as previously described [34]. Cells were then resuspended in a basic culture medium consisting of Dulbecco’s modified Eagle’s Medium (DMEM) (Life Technologies Grand Island, NY, USA; ref. 21,885–025) supplemented with 20% foetal bovine serum (FBS) (Life Technologies, Grand Island, NY, USA; ref. 10,270–106), 200 mM L-glutamine (Euroclone, Milan, Italy; ref. ECB3000D), and 200 U/mL penicillin 0. 1 mg/mL streptomycin (Euroclone, Milan, Italy; ref. ECB3001D). The culture medium was changed every 3 days. After reaching the confluence, cells were immunomagnetically separated using a primary monoclonal anti-c/kit (CD117) antibody (Miltenyi Biotec, Minneapolis, MN, USA) and labelled in the columns with a secondary antibody directly conjugated to MicroBeads (MACS Miltenyi Biotec, Bologna, Italy). Cells were then characterized by flow cytometry as previously described [34]. Briefly, cells were fixed at room temperature for 10 min using 1% formaldehyde and then permeabilized using a permeabilization buffer (eBioscienceMilano, Italy) for 30 min at 4°C. After a washing step, cells were incubated 1 h at 4°C, with primary antibodies directed against CD73, CD90 (BD Biosciences, San Jose, CA, USA), CD105 (Santa Cruz Biotechnology, Heidelberg, Germany), CD45 and CD31 (Sigma-Aldrich, Munich, Germany) and with fluorescein isothiocyanate (FITC)-conjugated secondary antibody for 1 h at 4°C in the dark. After washing, cells were analysed on a flow cytometer (CytoFlex, Beckman Coulter, Milan, Italy) by collecting 10,000 events. ADSCs positive for CD73, CD90 and CD105 at passage 5 were used for experiments. All experiments started at day 0, when cells reached the confluence and were performed twice (in three technical replicates) for a total of 21 days. Cells used as untreated control cells were maintained in normal growing medium (CTRL). A group of cells, used as positive control of adipogenic differentiation was cultured in a specific conditioned differentiation medium (DM) (StemPro Adipocyte Differentiation Medium, Gibco Life Technologies, Grand Island, NY, USA). Finally, a group of cells was cultured in DM in the presence of 10^−6^ M vitamin D (Sigma Aldrich Chemie GmbH, Munich, Germany, cod. C9765) (DM+VIT) or 5 mM metformin (Sigma Aldrich Chemie GmbH, Munich, Germany, cod. PHR1084) (DM+MET) or both (DM+VIT+MET).

### Gene expression analysis

Gene expression analysis was performed after 7, 14, and 21 d of culturing under the above described conditions. Approximately 1 µg of total RNA was extracted using the ChargeSwitch kit (Thermo Fisher Scientific, Grand Island, NY, USA; ref. CS14010) according to the manufacturer’s instructions, quantified by the NanoDrop™ One/OneC Microvolume UV-Vis spectrophotometer (Thermo Fisher Scientific, Grand Island, NY, USA) and reverse transcribed using the high-capacity cDNA reverse transcription kit (Thermo Fisher Scientific, Grand Island, NY, USA; ref.4368814). Real-time quantitative PCR was performed with Platinum® Quantitative PCR SuperMix-UDG Kit (Thermo Fisher Scientific, Grand Island, NY, USA; ref. 11,730–017) in triplicate using a CFX Thermal Cycler (Bio-Rad, Hercules, CA, USA). Amplification cycling was setted as specified in the protocol: 50°C for 2 min, 95°C for 2 min, and then cycled at 95°C for 15s, 55–59°C for 30s, and 60°C for 1 min, for a total of 40 cycles. Target Ct values of each sample were normalized to hGAPDH, which was considered as a reference gene. The relative values of the genes of interest were expressed as fold of change (2^−∆∆^Ct) of mRNA levels observed in undifferentiated ADSCs, used as control cells. The primers used (Thermo Fisher Scientific, Grand Island, NY, USA), are described in Table 1.

**Table 1.** Primers sequences.

Gene	Primer name	Forward	Reverse
*Glyceraldehyde-3-Phosphate Dehydrogenase*	*hGAPDH*	GAGTCAACGGAATTTGGTCGT	GACAAGCTTCCCGTTCTCAG
*adipocyte Protein 2*	*aP2*	AGACATTCTACGGGCAGCAC	TCATTTTCCCACTCCAGCCC
*lipoprotein lipase*	*LPL*	CAGGATGTGGCCCGGTTTAT	GGGACCCTCTGGTGAATGTG
*Acyl-CoA Thioesterase 2*	*ACOT2*	GAGGTCTTCACACTGCACCA	TCTTGGCCTCGAATGGTATC
*Uncoupling Protein 1*	*UCP1*	GTGGGTTGCCCAATGAATAC	TAAAAACAGAAGGGCGGATG

### Immunoblotting

Cells were cultured in the above described conditions for 21 days. Protein extraction was performed by RIPA Lysis and Extraction Buffer (Thermo Fisher Scientific, Grand Island, NY, USA; ref. 89,900) according to the manufacturer’s instructions and run by electrophoresis on 10% Novex Trisglycine polyacrylamide gels (Thermo Fisher Scientific, Grand Island, NY, USA; ref.NW00100BOX) in 4-morpholinepropanesulfonic acid, sodium dodecyl sulphate (MOPS SDS) Running Buffer (Thermo Fisher Scientific, Grand Island, NY, USA; ref. NP0001), using the XCell SureLock™ Mini-Cell (Thermo Fisher Scientific, Grand Island, NY, USA). Proteins were then transferred into polyvinylidene difluoride (PVDF) membranes (0.2 µm pore size) (Thermo Fisher Scientific, Grand Island, NY, USA; ref. IB301002) using iBlot® Dry Blotting System (Thermo Fisher Scientific, Grand Island, NY, USA). The membrane was saturated in non-fat dry milk (Bio-Rad Laboratories, California, USA; ref. 210,007,070) blocking buffer for 1 h at room temperature and incubated overnight in the presence of rabbit polyclonal anti-GAPDH(Santa Cruz Biotechnology, Texas, USA) and rabbit monoclonal anti-UCP1 (Cell Signalling, Massachusetts, USA) primary antibodies. At the end of incubation, membranes were washed and incubated with anti-rabbit peroxidase (HRP)-conjugated secondary antibody (Abcam, Cambridge, UK) for 2 h at RT. Protein expression was assessed by SuperSignal Chemiluminescent HRP Substrates (Thermo Fisher Scientific, Grand Island, NY, USA; ref. 34,096). Data from treated cells were reported as relative to the expression of untreated control cells and normalized to the expression level of GAPDH.

### Immunostaining

After 21 days of culture in the above described conditions, ADSCs were fixed for 30 min at RT with 4% paraformaldehyde (Sigma Aldrich Chemie GmbH, Germany; ref. 16,005) and permeabilized with 0.1% Triton X-100 (Thermo Fisher Scientific, Grand Island, NY, USA; ref. T8787)-PBS. Cells were then washed three times in PBS and incubated for 30 min with 3% bovine serum albumin (BSA)-0. 1% Triton X-100 in PBS (Thermo Fisher Scientific, Grand Island, NY, USA). A Double-Label Immunohistochemical procedure was carried out. Anti-Poly[ADP-ribose]polymerase (PARP1)(Cell Signalling, Massachusetts, USA), anti-transmembrane protein 26 (TMEM26) (Abcam, Cambridge, UK), proton-coupled amino acid transporter (PAT2) (Santa Cruz Biotechnology, Texas, USA), mTor (Abcam, United Kingdom) and cytochrome c (Cell Signalling, Massachusetts, USA) primary antibodies were incubated overnight at 4°C. At the end of incubation, cells were washed twice in PBS for 5 min and incubated with fluorescence-conjugated secondary antibodies (Life Technologies, USA) at 37°C for 1 h in the dark. Nuclei were labelled with 1 µg/mL 4,6-diamidino-2-phenylindole (DAPI) (Thermo Fisher Scientific, Grand Island, NY, USA). Fluorescence was acquired with a confocal microscope (TCS SP5, Leica, Nussloch, Germany).

### Statistical analysis

Statistical analysis was performed using GraphPad Prism 9.0 software (GraphPad, San Diego, CA, USA). The experiments were performed two times with three technical replicates for each treatment. For this study, Kruskal–Wallis rank sum, two-way analysis-of-variance ANOVA tests with Tukey’s correction and Wilcoxon signed-rank test were used, assuming a p value < 0.05 as statistically significant. We considered *p < 0.05, **p < 0.01, ***p < 0.001, ****p ≤ 0.0001.

## Results

### The combination of metformin and vitamin D modulates the expression of adipogenesis specific markers

**Figure 1.**
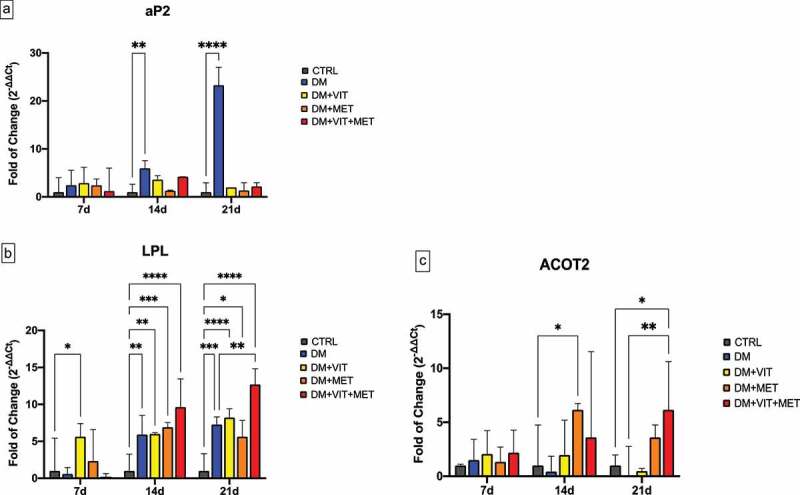
Expression of key adipogenesis regulatory genes. The expression of the adipogenesis orchestrating genes aP2 (Panel A), LPL (Panel B) and ACOT2 (Panel C) was evaluated after 7, 14 and 21 days in ADSCs cultured in the presence of adipogenic differentiation medium (DM) (blue bars), or in DM in the presence of vitamin D (DM+VIT) (yellow bars), or in DM in the presence of metformin (DM+MET) (Orange bars), or in DM with both metformin and vitamin D (DM+VIT+MET) (red bars), as compared to control untreated cells (grey bars). The mRNA levels for each gene were normalized to Glyceraldehyde-3-Phosphate-Dehydrogenase (GAPDH) and expressed as fold of change (2^−∆∆Ct^) of the mRNA levels observed in undifferentiated control ADSCs defined as 1 (mean ±SD; n = 6). Kruskal–Wallis rank sum, two-way analysis-of-variance ANOVA tests with Tukey’s correction and Wilcoxon signed-rank test were used. Data are expressed as mean ± SD referred to the control (* p ≤ 0.05; **p ≤ 0.01; ***p ≤ 0.001; ****p ≤ 0.0001).

Figure 1 shows the expression of key adipogenesis regulatory genes, adipocyte Protein 2 (aP2) (Panel A), Lipoprotein lipase (LPL) (Panel B) and acyl-CoA thioesterase 2 (ACOT2) (Panel C) in ADSCs cultured in the presence of different conditioned media. aP2 expression was significantly increased in cell exposed to differentiation medium alone (DM)(blue bar), while in the presence of the other conditioned media (DM+VIT; DM+MET; DM+VIT+MET) its expression was comparable to that observed in control untreated cells (Panel A), exhibiting an opposite trend to that observed for LPL and ACOT2 expression. In fact, LPL expression was induced in ADSCs cultured in the presence of different conditioned media after 14 days in culture (Panel B), being significantly increased in ADSCs cultured in the presence of both metformin and vitamin D after 21 days (DM+VIT+MET) (red bar), as compared to both control untreated cells and ADSCs cultured in the presence of DM alone. Similarly, ACOT2 expression (Panel C) was significantly upregulated in ADSCs exposed to metformin (DM+MET) (orange bar) after 14 days in culture and in ADSCs cultured in the presence of both metformin and vitamin D (DM+VIT+MET) (red bar) after 21 days, as compared to both control untreated cells and ADSCs cultured in the presence of DM alone.

### The combination of metformin and vitamin D induces the acquisition of a beige/brown adipogenic phenotype

**Figure 2.**
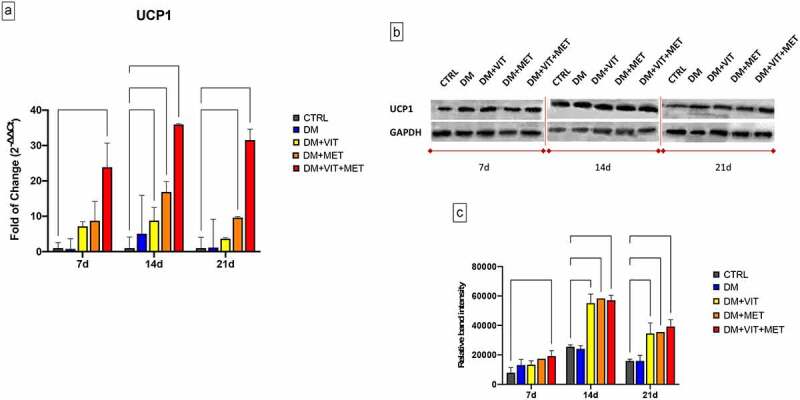
Expression of brown adipocyte specific UCP1 gene. A) The levels of UCP1 mRNA expression were evaluated after 7, 14 and 21 days in ADSCs cultured in the presence of adipogenic differentiation medium (DM) (blue bars), or in DM in the presence of vitamin D (DM+VIT) (yellow bars), or in DM in the presence of metformin (DM+MET) (Orange bars), or in DM with both metformin and vitamin D (DM+VIT+MET) (red bars), as compared to control untreated cells (grey bars). The mRNA levels were normalized to Glyceraldehyde-3-Phosphate-Dehydrogenase (GAPDH) and expressed as fold of change (2^−∆∆Ct^) of the mRNA levels observed in undifferentiated control ADSCs defined as 1 (mean ±SD; n = 6). Kruskal–Wallis rank sum, two-way analysis-of-variance ANOVA tests with Tukey’s correction and Wilcoxon signed-rank test were used. Data are expressed as mean ± SD referred to the control (* p ≤ 0.05; **p ≤ 0.01; ***p ≤ 0.001; ****p ≤ 0.0001). B) The protein levels were analysed after 7, 14 and 21 days by Western blot, using monoclonal antisera against UCP1 and GAPDH. The sizes of the bands were determined using pre-stained marker proteins. The data presented are representative of different independent experiments. C) Relative band intensity was measured with ImageJ software. Data are expressed as mean ± SD referred to the control (* p ≤ 0.05; **p ≤ 0.01; ***p ≤ 0.001; ****p ≤ 0.0001).

The presence of metformin alone (DM+MET) or in combination with vitamin D (DM+VIT+MET) in the adipogenic differentiation medium, was able to induce the acquisition of beige/brown phenotype, through the upregulation of Uncoupling Protein 1 (UCP1) (Figure 2). The gene expression of UCP1 was significantly increased in ADSCs exposed to DM+MET (orange bars) (* p ≤ 0.05) and to DM+VIT+MET (red bars) (***p ≤ 0.001) since the first days of culturing, as compared to both untreated controls and ADSCs cultured in the presence of DM alone (Panel A). Western blotting analysis confirmed this trend, showing higher protein levels in ADSCs exposed to DM+MET and DM+VIT+MET, as compared to untreated control cells (Panel B).

### Metformin and vitamin D orchestrate ADSC terminal differentiation

**Figure 3.**
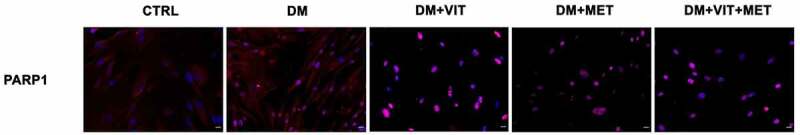
Immunohistochemistry analysis of adipogenic differentiation after 21 days in culture. Immunohistochemical analysis of the expression of PARP1 (AF594-labelled, red) was performed in ADSCs cultured in the presence of adipogenic differentiation medium (DM), or in DM in the presence of vitamin D (DM+VIT), or in DM in the presence of metformin (DM+MET), or in DM with both metformin and vitamin D (DM+VIT+MET), as compared to control untreated cells (CTRL). The figures are representative of different independent experiments. For each differentiation marker, fields with the highest yield of positively stained cells are shown. Relative intensity was measured with ImageJ software. Nuclei are labelled with 4,6-diamidino-2-phenylindole (DAPI, blue). Scale bars: 40 µm.

Immunohistochemical analysis confirmed that the presence of metformin alone or together with vitamin D is able to modulate ADSC adipogenic commitment (Figure 3). In particular, PARP1, clearly evident during adipocyte development, is significantly inhibited when ADSCs were cultured in the presence of metformin (DM+MET) or both metformin and vitamin D (DM+VIT+MET).

**Figure 4.**
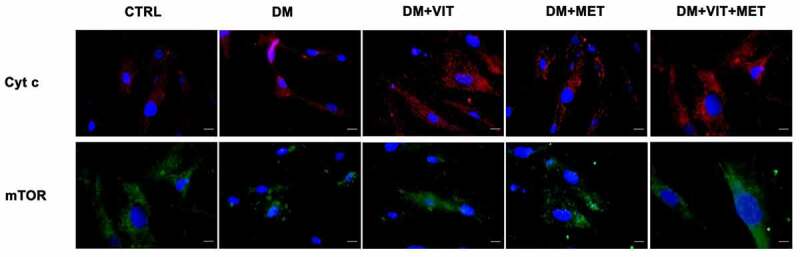
Immunohistochemistry analysis of adipogenic differentiation after 21 days in culture. Immunohistochemical analysis of the expression of Cyt c (AF594-labelled, red) and mTor (AF488-labelled, green) was performed in ADSCs cultured in the presence of adipogenic differentiation medium (DM), or in DM in the presence of vitamin D (DM+VIT), or in DM in the presence of metformin (DM+MET), or in DM with both metformin and vitamin D (DM+VIT+MET), as compared to control untreated cells (CTRL). The figures are representative of different independent experiments. For each differentiation marker, fields with the highest yield of positively stained cells are shown. Relative intensity was measured with ImageJ software. Nuclei are labelled with 4,6-diamidino-2-phenylindole (DAPI, blue). Scale bars: 40 µm.

At the same time, also cytochrome c and mTor expression was significantly increased when ADSCs were cultured in the presence of vitamin D (DM+VIT) or metformin (DM+MET) or both metformin and vitamin D (DM+VIT+MET), while it was poorly expressed in ADSCs cultured in adipogenic differentiation medium alone (DM)(Figure 4).

**Figure 5.**
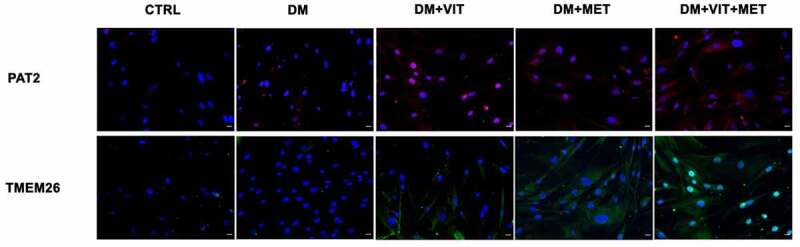
Immunohistochemistry analysis of adipogenic differentiation after 21 days in culture. Immunohistochemical analysis of the expression of PAT2 (AF594-labelled, red) and TMEM26 (AF488-labelled, green) were performed in ADSCs cultured in the presence of adipogenic differentiation medium (DM), or in DM in the presence of vitamin D (DM+VIT), or in DM in the presence of metformin (DM+MET), or in DM with both metformin and vitamin D (DM+VIT+MET), as compared to control untreated cells (CTRL). The figures are representative of different independent experiments. For each differentiation marker, fields with the highest yield of positively stained cells are shown. Relative intensity was measured with ImageJ software. Nuclei are labelled with 4,6-diamidino-2-phenylindole (DAPI, blue). Scale bars: 40 µm.

On the other hand, PAT2 and TMEM26 expression (Figure 5) was increased in cells exposed to differentiation medium in the presence of vitamin D (DM+VIT) or metformin (DM+MET) or both (DM+VIT+MET), while it was completely undetectable in ADSCs cultured in adipogenic differentiation medium alone (DM).

## Discussion

ADSCs are mesenchymal stem cells with a great plasticity for application in regenerative medicine **[**39**]**. Adipogenesis is a complex multi-step process finely regulated by genes, signalling pathways and epigenetic modifications **[**40**]**. We have recently demonstrated that different bioactive molecules can be used in the attempt to counteract ADSC adipogenic differentiation and lipid accumulation in the adipose tissue. For example, the combination of melatonin and vitamin D was able to induce the appearance of osteogenic phenotype by inhibiting the PPAR-γ expression and activating the epigenetic modulators SIRT and HDAC **[**33,34**]**. In addition, also metformin, well known for inducing weight loss in overweight or obesity patients **[**41**]**, when combined with vitamin D, modulates the expression of CYP450 enzymes and miRNAs, blocking adipogenesis **[**37**]**. It has been demonstrated that vitamin D plays a key role in the regulation of metabolism, modulating white adipocyte differentiation and energy expenditure **[**42,43**]**. Vitamin D deficiency has been linked to oxidative stress, inflammation, ageing, cardiovascular disease, and diabetes. Optimal serum levels of vitamin D are ≥ 30 ng/mL, while levels between 20–30 ng/mL indicate ‘insufficiency’, and levels < 20 ng/mL indicate ‘deficiency’ **[**44**]**. Vitamin D influences most of the risk factors and molecular mechanisms associated with cerebrovascular disease, preventing their onset, progression, and severity **[**45**]**. In addition, supplementation in patients with T2DM under standard metformin therapy attenuates the risk of oxidative stress, metabolic syndrome and related cardiovascular events **[**46,47**]**. Moreover, 1,25(OH)_2_D_3_/VDR signalling suppressed differentiation of 3T3-L1 white adipocytes together with increased expression of uncoupling proteins (Ucp1 and Ucp2) and development of BAT **[**48**]**. Within this context, we previously evaluated the ability of the two molecules to modulate adipogenic differentiation, finely tuning the inflammatory response, cytokines secretion and autophagy **[**38**]** In the present paper we evaluated for the first time, the effect of these molecules in inducing a different phenotype during ADSC adipogenic differentiation. In addition to white and brown adipose tissue, beige or brite adipocytes also have a role in body glucose regulation and thermogenesis **[**49**]**. These ‘brown-like’ cells express an increased mitochondrial activity and oxidative metabolism and high levels of UCP1 and other fat specific markers, as PAT2 and TMEM 26 **[**26,27,50**]**. mTOR, is a Ser/Thr protein kinase regulating protein and lipid synthesis, cell proliferation and metabolism, and autophagy **[**51**]**. Recently, mTOR-related signalling pathways have been reported to play pivot roles in the regulation of adipose tissue browning and chemical energy dissipation through thermogenesis **[**52**]** but the precise mechanisms are still poorly understood. Inhibition of mTOR completely blocks BAT expansion, reducing oxygen consumption and mitochondrial biogenesis **[**53**]**. Indeed, as already shown by other authors, the mTOR pathway is crucial for the early stages of brown preadipocytes differentiation, enhancing glucose uptake through the GLUT1 transporter **[**52**]**. Our results show that metformin, alone or in combination with vitamin D, is able to induce mTOR expression, probably activating AMPK, which in turn also induces increased expression of UCP1.

Adipocytes terminal differentiation is characterized by increased expression of PPARγ and PARP1 proteins and lipid accumulation **[**28**]**. In particular, PARP1 knockout mice, show a reduced lipid deposition with induction of UCPs, and increased energy expenditure **[**54**]**. Deletion or pharmacological inhibition of PARP1 supports mitochondrial biogenesis and function, providing protection against metabolic disease **[**55**]**. According to other Authors’ findings obtained in knockout mice, our results show that ADSC exposure to metformin, alone or in combination with vitamin D, was able to downregulate PARP1 expression, increasing thermogenic activity through upregulation of mitochondrial UCP1, PAT2 and TMEM26 **[**28,56**]**. Furthermore, several drug can be used for their anti-obesity effects **[**57**]**. Some studies demonstrated the effect of beta(3)-adrenergic agonist in decreasing expression levels of aP2 and PPARγ, affecting fat deposition in WAT and promoting thermogenesis **[**58**]**. The same effect was observed by other Authors in 3T3-L1 adipogenesis after treatment with raspberry ketone, that revealed the inhibition of adipogenic markers by the proper regulation of autophagy **[**59**]**.

Here, we observed a downregulation of aP2 in the presence of metformin and vitamin D, accompanied by an induction of thermogenic protein UCP1 and browning differentiation. Opposite trend what observed in LPL and ACOT2 expression. These genes were significantly upregulated when cells were cultured in the presence of metformin alone or in combination with vitamin D. In fact, regulation of mitochondrial ACOT2 occurs mainly in the late phase of adipocyte differentiation, and its expression increases with increasing of β-oxidation **[**60,61**]**. ACOT2 expression may be upregulated in brown adipocyte to support the fat storage and thermogenic activity of this cells **[**15**]**. LPL is a major triglyceride transporter and increases lipid absorption **[**9**]**. In white adipose tissue, increased LPL activity is related with increased fat mass, chronic inflammation and insulin resistance **[**62,63**]**. Conversely, in brown adipose tissue LPL upregulation exerts positive effects on metabolic disease by burning excess calories through activation of mitochondrial thermogenesis **[**64**]**. Human studies currently show that activation of thermogenic adipose tissue is associated with a small and negligible loss of fat mass **[**65**]**. Moreover, no dietary intervention has yet been shown to affect Ucp1 expression, which is only expressed at extremely low levels in subcutaneous fat depots **[**66,67**]**. Several authors demonstrated the effect of 5 mM metformin in the modulation of cell behaviour **[**68–70**]**, as well as of vitamin D, involved in osteogenic differentiation **[**34,71**]**. The combination of the two molecules could therefore open new therapeutic approaches for *in vivo* evaluation of the physiological response. Our results describe for the first time the ability of metformin and vitamin D to promote ADSC differentiation towards beige phenotype, suggesting their possible direct application in prevention of obesity and metabolic disorders, driving adipogenesis towards metabolically active brown adipose tissue.

## Data Availability

The authors confirm that the data supporting the findings of this study are available within the article [and/or] its supplementary materials (http://dx.doi.org/10.1080/21623945.2022.2085417).
